# Vacuum-assisted closure therapy for an empyema with bronchopleural fistula with artificial dermis covering the bronchial stump

**DOI:** 10.1186/s44215-023-00051-4

**Published:** 2023-06-14

**Authors:** Akira Matsumoto, Tsuyoshi Shoji, Hiromichi Katakura

**Affiliations:** Department of Thoracic Surgery, Otsu Red Cross Hospital, 1-1-35, Nagara, Otsu, Shiga Japan

**Keywords:** Empyema, Bronchopleural fistula, Vacuum-assisted closure therapy, Open window thoracotomy, Artificial dermis

## Abstract

**Background:**

Vacuum-assisted closure (VAC) therapy has recently been reported to be useful for treating empyema complicated by bronchopleural fistula (BPF). Since the pleural cavity must be closed to apply this treatment, various fistula closure methods for the fistula have been used in previous reports. We report a case in which VAC therapy could be applied more safely by simply covering the fistula with an artificial dermis.

**Case presentation:**

A 72-year-old man who underwent thoracoscopic lower lobectomy of the right lung for squamous cell carcinoma pT1cN0 developed BPF on the postoperative day 22. First, infection control was achieved by open-window thoracostomy and antibiotic therapy. Subsequently, VAC therapy was applied by covering the fistula with an artificial dermis, and closure of the bronchial stump was completely achieved. Subsequently, the thoracostomy was closed surgically.

**Conclusion:**

Covering the fistula with an artificial dermis allowed for the introduction of VAC therapy when treating empyema complicated by BPF.

## Background

Postoperative empyema associated with bronchopleural fistula (BPF) is life-threatening and difficult to manage. As indicated by several studies [1], the treatment of this complication often begins with infection control through an open-window thoracostomy (OWT) to drain the pleural cavity. Once the infection is improved, there are several options for the closure of the fistula and the thoracostomy. Recent reports have shown that vacuum-assisted closure (VAC) therapy may be useful [2]. As it is necessary to seal the cavity to introduce this therapy, several techniques to close the fistula are reported [3, 4]. We report a case of empyema complicated by BPF in which VAC therapy was effective by simply covering the fistula with an artificial dermis.

## Case presentation

**Fig. 1:**
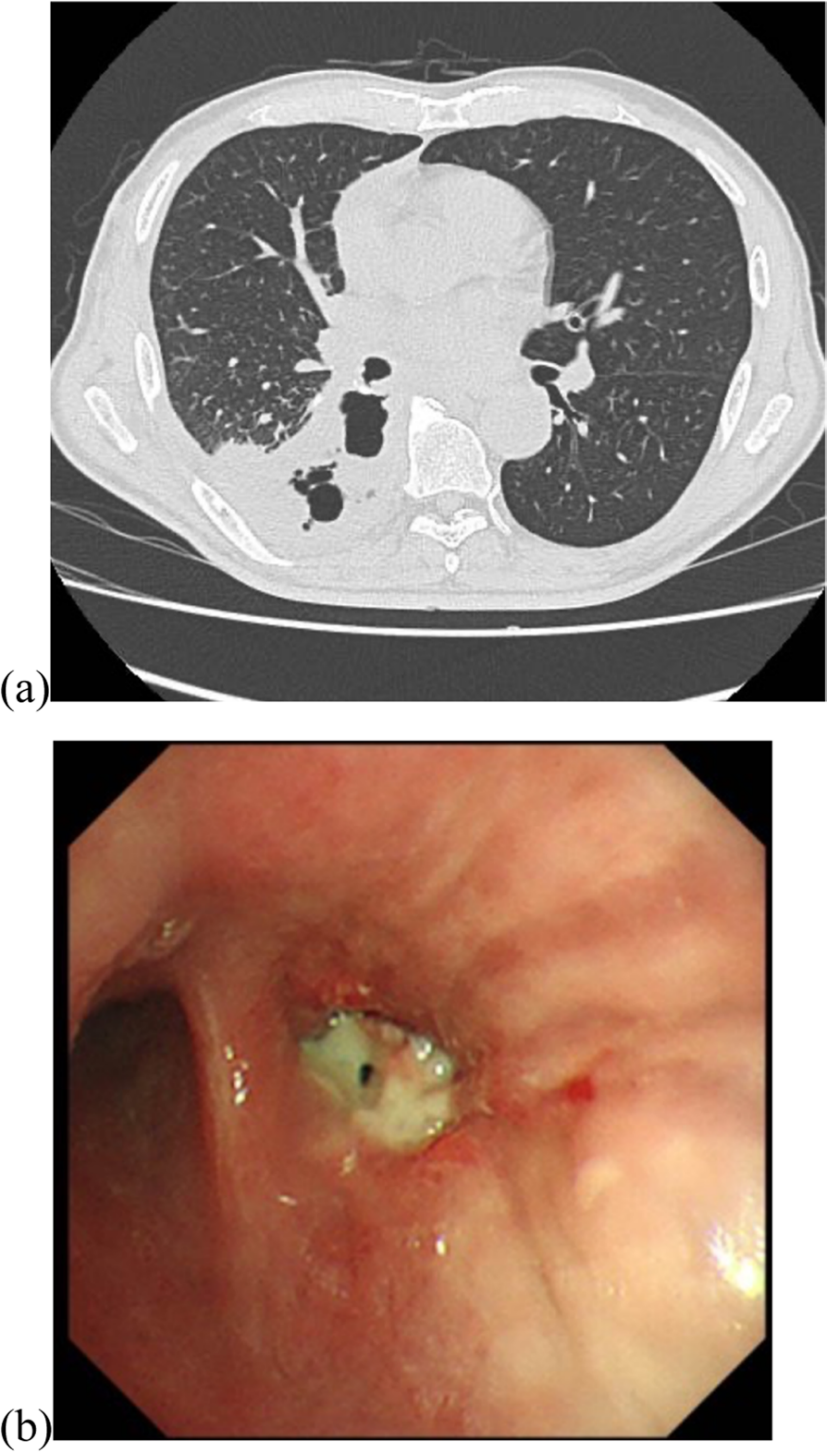
CT scan of the chest (**a**) and bronchoscopy (**b**) showing the fistula between the bronchial stump and the pleural cavity. CT, computed tomography

A 72-year-old man underwent a video-assisted thoracoscopic right lower lobectomy for a pT1cN0 lung squamous cell carcinoma. No reinforcement was used for the bronchial stump because he had no nonsurgical risk factor of BPF. Two weeks post-surgery, the patient was readmitted with worsening chest pain and was diagnosed with postoperative pleurisy. After drainage and antibiotic treatment, the signs of infection tended to improve; however, on postoperative day 22, continuous air leakage suddenly appeared. Flexible bronchoscopy and computed tomography (CT) revealed that the bronchial stump of the lower bronchus was opened and connected to the pleural cavity (Fig. 1).

**Fig. 2:**
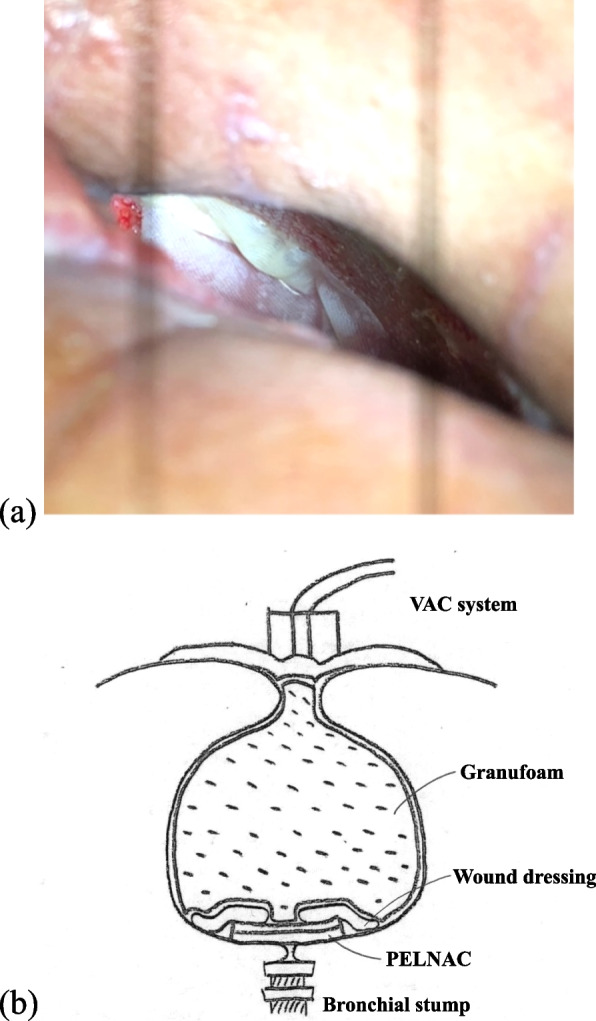
**a** The artificial dermis (PELNAC Gplus, Gunze, Kyoto, Japan) covers the fistula. **b** This is an illustration of how to introduce VAC therapy. The fistula is covered with PELNAC, around which is covered with wound dressings, and the thoracic cavity is filled with Granufoam, sealed with plastic film, and connected to the VAC system

**Fig. 3:**
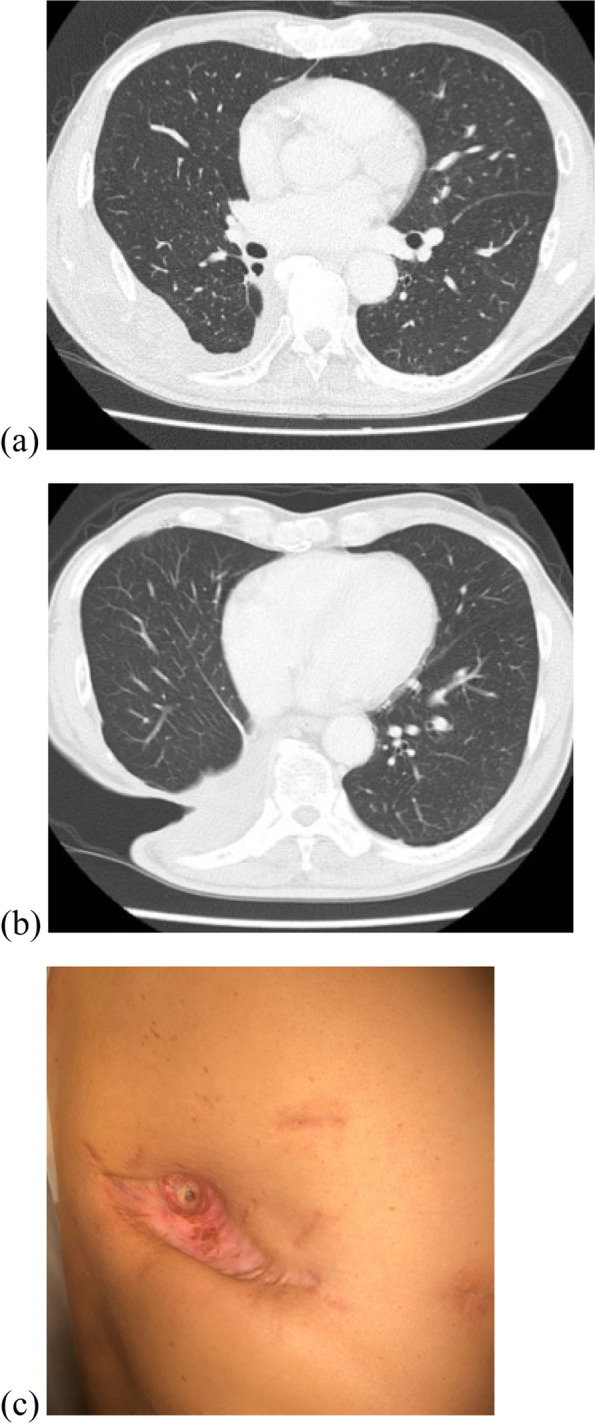
**a**, **b** CT scan of the thorax shows the expansion of the residual lung, and there are no signs of the fistula. **c** Appearance of the right lateral chest. The bottom of the open-window thoracostomy was observed

**Fig. 4:**
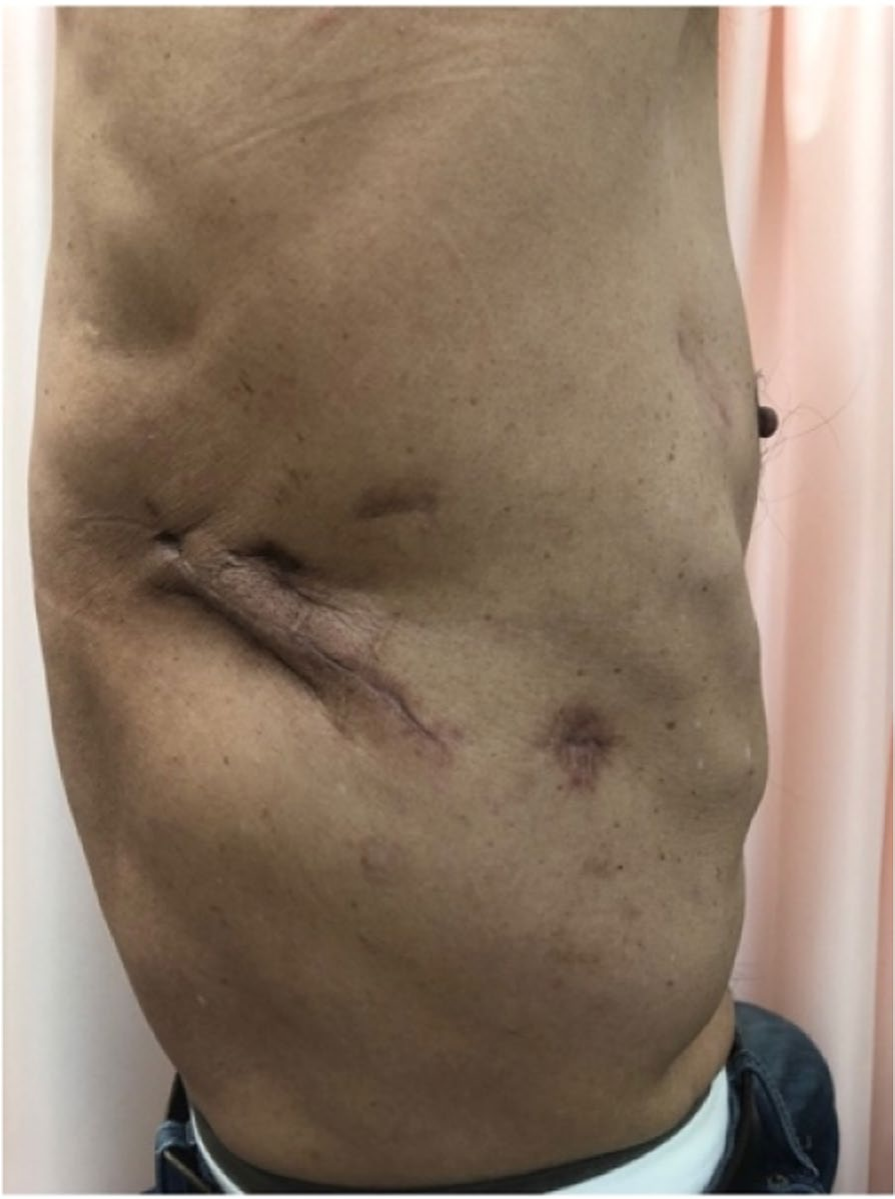
The appearance of the right lateral chest after thoracoplasty. Lateral thoracic depression was smoothed by resecting the posterolateral segments of the ninth rib and transposition the connective and muscular tissue into the depression

Since the patient did not accept the choice of primary closure of the BPF by an omental flap transposition, OWT was performed by resecting the posterolateral segments of the seventh and eighth ribs, and gauze exchange and antibiotic treatment were continued. Four weeks after surgical debridement, the infection improved, the thoracic cavity began to be replaced by granulation tissue, and the bronchial fistula and the pleural space showed narrowing; however, spontaneous closure was considered to take too long. VAC therapy (V.A.C. Therapy, KCI, San Antonio, TX, USA) was introduced by covering the fistula with an artificial dermis (PELNAC Gplus, Gunze, Kyoto, Japan) (Fig. 2). The bronchial stump was observed as a 1 cm slit and 3S size (40 × 30 mm) of PELNAC was large enough to cover it. The area around the artificial dermis was reinforced with wound dressings, and the pleural cavity was filled with Granufoam (V.A.C. Granufoam Dressing, KCI, San Antonio, TX, USA) and covered with plastic film, and suction at − 25 mmHg was administered for 4 weeks. First, the intermittent mode of 1-min suction and 1-min pause was used, and once airtightness was achieved, the continuous suction mode was used. Even after the removal of the reinforcement film of the artificial dermis 3 weeks after introduction, it was possible to maintain airtightness if the tissues were attached to each other. Owning to insurance limitations in Japan, negative pressure closure therapy was continued using a chest tube and drainage bag. A chest tube was placed in the intrathoracic cavity, filled with gauze, sealed with surgical drapes, and connected to a chest drainage bag. Airtightness was maintained at any time, and the fistula was completely closed. Continuous suction at − 20 cmH2O was continued for 3 weeks, after which the patient was discharged (Fig. 3). The patient continued to be followed up in an outpatient clinic without recurrence of the infection. Approximately 1 year after onset, the wound was closed by thoracoplasty for cosmetic reasons (Fig. 4).

## Discussion

Recently, VAC therapy was reported to be effective in the treatment of empyema with BPF. VAC therapy applies negative pressure to promote wound healing and expansion of the remaining lung. Owning to the mechanism of the treatment, the cavity must be sealed to carry out the therapy. Therefore, VAC therapy is often applied for the closure of the OWT after the fistula is closed using surgical or endoscopic methods.

In this case, VAC therapy could be applied by simply covering the fistula with an artificial dermis, a general artificial material composed of an inner layer of collagen sponge and an outer silicone layer. It has been used by plastic surgeons to treat skin defects [5]. Covering the BPF with an artificial dermis allowed the pleural cavity to remain airtight by the outer layer, and the inner layer was thought to be the starting point for BPF healing. This treatment was considered ethically acceptable because the thoracic cavity replaced by granulation tissue can be viewed as an extensive skin defect, and the patient preferred to avoid surgical closure. To the best of our knowledge, this is the first report of case with BPF treated with an artificial dermis for VAC therapy.

Surgical or endoscopic closure of the BPF is a high-risk and high-return method. If it responds, a quick cure can be expected; however, if it does not, the situation may worsen. Re-dissection after surgical closure may result in additional injury of the bronchial stump, and the free tissue used is no longer available, thus limiting the next treatment options. If the endoscopic filling has dropped out, the BPF may be enlarged. In this respect, our treatment may be safer and does not limit the treatment options. Even if complete closure of the BPF is not achieved, it may be possible to switch to surgical or endoscopic closure treatment under better situations.

Certainly, there are some limitations to this treatment. If the infection is not improved, no therapeutic effect is expected and it could make the infection worse. It can take an enormous amount of time, depending on the patient’s nutritional status and the size of the bronchial stump. Furthermore, in this case, it took multiple attempts to place the artificial dermis so that the chest cavity was sealed, and initially, negative pressure had to be minimized as much as possible so that the placed dermis did not lift up. However, if it works, the benefit of avoiding surgical or endoscopic closure is significant.

Overall, VAC therapy with an artificial dermis helped close the BPF, in our case. We believe that this treatment could be safer and less invasive than conventional surgical or endoscopic closure methods and could be used to treat BPFs.

## Data Availability

Data supporting the study’s findings are available from the corresponding author upon reasonable request.
